# Neoadjuvant Chemotherapy vs Chemoradiotherapy for Malignancy of Esophagus: A Prospective Comparative Study

**DOI:** 10.7759/cureus.73525

**Published:** 2024-11-12

**Authors:** Vaibhav K Varshney, Vishu Jain, B Selvakumar, Subhash Soni, Peeyush Varshney, Lokesh Agarwal, Sunita Suman, Bharti Varshney, Sabir Hussain, Akhil Dhanesh Goel, Puneet Pareek, Poonam Elhence

**Affiliations:** 1 Surgical Gastroenterology, All India Institute of Medical Sciences, Jodhpur, Jodhpur, IND; 2 Pathology, All India Institute of Medical Sciences, Jodhpur, Jodhpur, IND; 3 Gastroenterology, Dr. Sampurnanand Medical College, Jodhpur, IND; 4 Community Medicine & Family Medicine, All India Institute of Medical Sciences, Jodhpur, Jodhpur, IND; 5 Radiation Oncology, All India Institute of Medical Sciences, Jodhpur, Jodhpur, IND

**Keywords:** complication, minimally invasive esophagectomy, neoadjuvant chemoradiotherapy, neoadjuvant chemotherapy (nact), pathological response rate, squamous cell carcinoma esophagus, survival analysis

## Abstract

Introduction: Neoadjuvant chemoradiation (NACRT) followed by surgery has become the standard of care for esophageal squamous cell carcinoma (ESCC). This study compared the tolerability and oncological benefit of neoadjuvant chemotherapy (NACT) with those of NACRT for the treatment of ESCC.

Methods: A prospective quasi-experimental comparative study was conducted from July 2019 to August 2023 to assess the efficacy of the NACT regimen of two cycles of paclitaxel and carboplatin as an alternative to standard NACRT. Either NACT or NACRT was given to patients with resectable ESCC (clinical stage IB-IIIC), after which they underwent minimally invasive esophagectomy with two-field lymphadenectomy. Radiological and pathological responses to neoadjuvant therapy, perioperative morbidity, mortality, and recurrence-free and overall survival rates were compared.

Results: Out of the 74 patients enrolled, 63 were included in the study after exclusion. Of these, 30 received NACT, and 33 received NACRT. The baseline demographics, tumor characteristics, incidence of neoadjuvant therapy-related adverse events, and perioperative morbidity were comparable between the two groups. The median number of lymph nodes retrieved (21 vs 19, p=0.19) and R0 resection rate (100% vs 94%) were similar. Although the pathological response was significantly better in the NACRT arm, at a median follow-up of 32.5 (20.75-48) months, there was a non-significant trend toward better recurrence-free survival in the NACRT group (57 vs 36 months, p-value - 0.831), with median overall survival yet to be achieved in both groups.

Conclusion: Compared with NACRT, NACT for ESCC is well tolerated and has non-inferior oncological outcomes. NACT could be a feasible alternative to NACRT in such patients, especially if the radiotherapy option is not feasible or available.

## Introduction

Neoadjuvant chemoradiation (NACRT) followed by surgery has become the optimal treatment for carcinoma of the esophagus after the results of the ChemoRadiotherapy for Oesophageal Cancer followed by Surgery Study (CROSS) [1]. However, this study only advocated the superiority of NACRT followed by surgery over surgery alone and did not suggest that NACRT is superior to neoadjuvant chemotherapy (NACT) for malignancy of the esophagus.

Importantly, with radical two-field esophagectomy, the primary tumor bed is treated twice, first with NACRT, followed by radical surgery. Additionally, NACRT causes significant perioperative morbidity in the form of increased intraoperative difficulty, pulmonary and cardiac complications, and perioperative mortality [2-6]. Clinical trials have attempted to deduce the superiority of NACRT over NACT, with variable results and no definite conclusions. Furthermore, studies have been conducted on esophageal cancer overall, including both squamous cell carcinoma (SCC) and adenocarcinoma, making the results heterogeneous [2-6]. Esophageal SCC (ESCC) and adenocarcinoma are distinctive diseases with different prognoses and should be approached separately.

The next query concerns the best chemotherapy regimen in the neoadjuvant setting for optimal systemic response. Various NACT regimens have been studied for the treatment of esophageal carcinoma, such as CF (cisplatin with 5-fluoro-uracil), FLOT (5-fluorouracil, leucovorin, oxaliplatin, and docetaxel), ECF (epirubicin, cisplatin, and 5-fluorouracil), and PC (paclitaxel and carboplatin) [7-9]. However, there is no definitive answer regarding which is the superior regimen. Hence, the appropriate NACT regimen for mid and lower thoracic ESCC remains to be confirmed.

These conflicting results led us to perform a prospective quasi-experimental study comparing NACT with NACRT for SCC of the esophagus or esophagogastric junction (OGJ). This study was conducted with the premise that NACT followed by minimally invasive transthoracic esophagectomy (MITE) with two-field lymphadenectomy should be sufficient treatment for ESCC. We also aimed to study which neoadjuvant therapy has a better safety profile and tolerability in the Indian population.

This article was previously posted to the Research Square preprint server on May 28, 2024.

## Materials and methods

A prospective quasi-experimental comparative study was conducted in the Department of Surgical Gastroenterology and Radiotherapy at the All India Institute of Medical Sciences (AIIMS), Jodhpur, Rajasthan, India, from July 2019 to June 2022, and the recruited patients were followed up until August 2023. The study protocol was approved by the Institute Review Board and Ethics Committee of AIIMS (AIIMS/IEC/2019-20/863).

Patient criteria

All patients aged 18-75 years with an Eastern Cooperative Oncology Group (ECOG) performance status of 0-2, histologically proven SCC mid-lower thoracic esophagus or OGJ and clinical-stage IB-IIIC SCC according to the American Joint Committee on Cancer 8th Edition, 2016 (cT1-4a, any N (except T1N0) or N+, any T or tumor length > 3 cm) were included. Patients who had a previous history of receiving chemotherapy or radiotherapy, adenocarcinoma on histology, metastatic disease, medical conditions precluding the administration of chemotherapy or radiotherapy, pregnancy/nursing status, and preexisting peripheral neuropathy were excluded.

Sample size calculation

Klevebro et al. reported a 38% surgical complication rate in the NACRT group compared with 35% in the NACT group [10]. A sample size calculation was performed to assess noninferiority, i.e., NACT is not worse than NACRT. If there was a true difference in favor of NACRT of 3% (38% vs 35%), then 62 patients would be required (31 per group) to be 80% sure that the upper limit of a one-sided 95% confidence interval would exclude a difference in favor of NACRT of more than 35% (noninferiority limit), with consideration for a 10% contingency for dropouts. A total of 74 patients were enrolled.

Intervention

Patients were evaluated via upper gastrointestinal endoscopy (UGIE) and biopsy. Baseline staging was performed via contrast-enhanced computed tomography (CECT) of the neck, thorax, and abdomen or positron emission tomography with 2-deoxy-2-(fluorine-18) fluoro-D-glucose integrated with CT (F-18 FDG PET-CT). Patients were informed about both NACT and NACRT in detail, including the duration of therapy, toxicity, and interval between the completion of neoadjuvant treatment and surgery. Patients were then given either NACT or NACRT, as in a quasi-experimental study, based on patient choice and radiotherapy waitlist time.

NACT Regimen

Two cycles of paclitaxel and carboplatin were given 21 days apart. A paclitaxel dose of 135-200 mg/m^2^ was given as a three-hour infusion, followed by carboplatin dosed to an area under the curve (AUC) of 6 by Calvert’s formula, given as a one-hour infusion. All patients were premedicated with dexamethasone 16 mg, given intravenously before each dose of chemotherapy, and with other medications as per the standard protocol. On day 22, if the total leucocyte count (TLC) was lower than 1000 per microliter or the platelet count was lower than 50000 per microliter, administration of the next cycle was delayed by seven days until the recovery of these blood parameters. Symptomatic management postchemotherapy was given as per the standard of care. The toxicity profile was noted every week as per the Common Terminology Criteria for Adverse Events (CTCAE) after each cycle of chemotherapy or if a patient presented with side effects of treatment [11].

NACRT Regimen

Carboplatin (AUC 2 mg/mL per minute) and paclitaxel (50 mg/m^2^ body surface area) were given for five cycles on days 1, 8, 15, 22, and 29. A concurrent radiation dose of 41.4 Gray (Gy) was given in 23 fractions of 1.8 Gy for five days per week, starting on the first day of the first chemotherapy cycle. The radiation field included the primary tumor with a 4 cm cranio-caudal extension with a 1 cm lateral margin and potential metastatic lymph nodes on the basis of preoperative radiological imaging. On days 8, 15, 22, or 29, if the TLC was lower than 1000 per microliter or the platelet count was lower than 50,000 per microliter, the next cycle of NACRT was delayed by one week until these parameters were recovered.

Furthermore, in the case of oral ulcers, if there was recurrent vomiting despite antiemetic premedication, NACRT was delayed by one week. Further chemotherapy was withheld in patients with febrile neutropenia (defined as a neutrophil count <500 per microliter and a body temperature >38.5°C) and persistent creatinine clearance of <50%. During NACRT, laboratory tests (hemogram and serum creatinine) were performed weekly, whereas radiological assessments were performed only if indicated. The toxicity profile was noted by CTCAE after each cycle of chemotherapy [11].

Response assessment

Response assessment was carried out four to six weeks after the completion of NACT or NACRT with an F-18 FDG PET‒CT scan and UGIE. All patients with resectable esophageal cancer without systemic dissemination on post-neoadjuvant treatment staging were considered for surgical exploration. Patients underwent resection four to six weeks after the completion of neoadjuvant treatment. They were prehabilitated for five to seven days before surgery via incentive spirometry, smoking cessation, optimization of comorbidities, and high-protein oral/enteral nutritional supplementation.

Surgery

A thoracoscopic or robot-assisted (da Vinci Xi, Intuitive Surgical, Sunnyvale, USA) mobilization of the thoracic esophagus with total thoracic lymphadenectomy, laparotomy with suprapancreatic retroperitoneal lymphadenectomy and gastric conduit formation, and cervical esophagogastric anastomosis (McKeown procedure) were performed. Postoperatively, the patients were managed via enhanced recovery after surgery protocols.

Outcomes

Perioperative morbidity in terms of both surgical and nonsurgical complications was recorded. Surgical complications included anastomotic leakage, conduit necrosis, bleeding, chylothorax, and recurrent laryngeal nerve palsy. Nonsurgical complications included respiratory complications such as pneumonia, pleural effusion, and respiratory failure, and cardiac complications such as arrhythmias, myocardial infarction, and embolism. The severity of the complication was scored according to the Clavien‒Dindo system for classifying surgical complications [12]. The 30-day and 90-day readmission, reoperation, and mortality rates, and their causes were recorded. The pathological response was analyzed as per the modified Ryan scheme in the College of American Pathologists (CAP) cancer protocol for esophageal carcinoma [13]. Microscopically, radical resection (R0) was defined as a tumor-free resection margin of at least 1 mm.

Follow-up

Patients were followed up at three-month outpatient visits with detailed clinical assessment regarding any symptoms or weight loss. A surveillance F-18 FDG PET‒CT scan was performed every six months for the first two years and then annually to look for recurrence. UGIE and neck/chest/abdominal CT scans were performed when clinically indicated. Overall survival (OS) was defined as the time from enrollment into the study to all-cause death, whereas recurrence-free survival (RFS) was defined as the time from enrollment into the study to disease recurrence during follow-up investigations. Patients who underwent R1 resection, had positive neck lymph nodes, or had recurrent disease were given palliative treatment or best supportive care as per multidisciplinary tumor board meetings and/or patient wishes.

Statistical analysis

The data were analyzed according to the per-protocol principle for all patients. Categorical data are presented as counts and percentages and were compared via the chi-square test and Fisher’s exact test. Continuous nonparametric data are presented as a median with an interquartile range (IQR) and were compared via the Mann‒Whitney U test. RFS and OS were estimated via the Kaplan‒Meier method. The influence of variables on survival was analyzed via the log-rank test. The statistical calculations were performed via the IBM SPSS Statistics for Windows, Version 23.0 (Released 2015; IBM Corp., Armonk, New York, United States). Statistical significance was considered at a p-value of 0.05 or less.

## Results

Baseline characteristics

**Figure 1:**
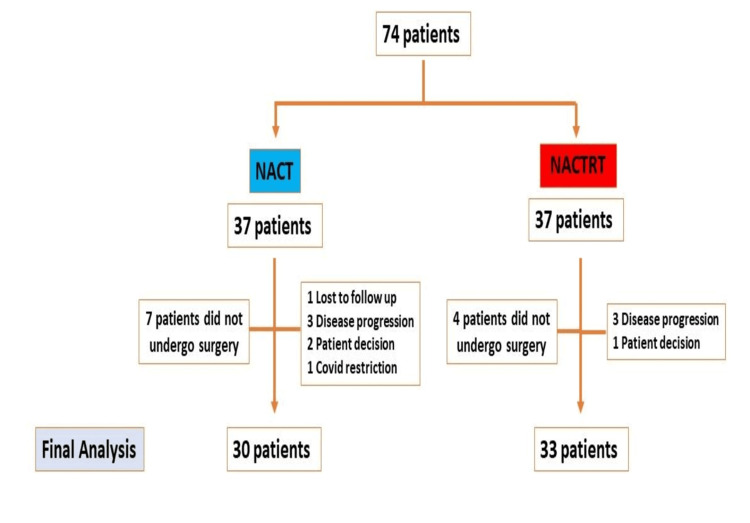
CONSORT diagram CONSORT: Consolidated Standards of Reporting Trials; NACT: neoadjuvant chemotherapy; NACRT: neoadjuvant chemoradiation

Among the 74 patients enrolled, 63 completed neoadjuvant treatment followed by radical MITE and were hence included in the study (Figure 1). Among these patients, 30 received NACT, and 33 received NACRT.

Both NACT and NACRT groups were comparable in terms of baseline demographic, clinical, and tumor characteristics. The clinical (c) T3 and cN+ stages were the most common in both groups (Table 1).

**Table 1:** Comparison of baseline variables in esophageal squamous cell carcinoma patients receiving NACT and NACRT ^*^Modified Takita Grading; ^a^ Mann Whitney U test; ^b^ Chi-Square test ECOG: Eastern Cooperative Oncology Group; NACT: neoadjuvant chemotherapy; NACRT: neoadjuvant chemoradiotherapy; IQR: interquartile range; TLC: total leucocyte count

Parameters	NACT (n=30)	NACRT (n=33)	p-value
Age (years), median (IQR)	51.5 (44-66)	54.0 (43-60)	0.549^a^
Gender (female), n (%)	13 (43)	22 (67)	0.063^b^
Grade of dysphagia*, median (IQR)	3 (2-4)	3 (2-4)	0.640^a^
ECOG performance status, 0/1/2, n (%)	14 (46.7)/ 10(33.3)/ 6(20.0)	18 (54.5) /12 (36.4) / 3(9.1)	0.462^b^
Hypertension n (%)	1 (3.3)	1 (3.0)	0.945^b^
Diabetes Mellitus n (%)	1 (3.3)	2 (6.0)	0.611^b^
Haemoglobin (g/dl), median (IQR)	11.5 (10.4-12.6)	11.8 (10.8-12)	0.962^a^
TLC (x 10^3^/μl), median (IQR)	6.0 (5.0-7.6)	5.8 (4.8-7.3)	0.437^a^
Platelet (x 10^3^/μl), median (IQR)	246 (161-290)	227 (198-254)	0.386^a^
Albumin (g/dl), median (IQR)	3.8 (3.5-4.0)	3.7 (3.5-3.9)	0.799^a^
Location of tumor Lower 1/3^rd^/Middle 1/3^rd^, n (%)	18 (60)/12 (40)	13 (39.4)/20(60.6)	0.102^b^
Clinical tumor stage (cT), 1/2/3/4, n (%)	1 (3)/ 8(27)/15(50)/ 6(20)	3 (9)/ 7(21)/16 (49)/ 7(21)	0.792^b^
Clinical nodal stage (cN), N0 / N+, n (%)	6 (20) / 24(80)	11(33) / 22(67)	0.234^b^

Clinical and radiological response and adverse effects of neoadjuvant therapy

The clinical median improvement in the grade of dysphagia after neoadjuvant therapy was comparable in both groups. Compared with the NACT group, the NACRT group had a better radiological response at the primary site and a nonsignificant trend toward better response at the nodal site and cT and cN downstaging. The incidence of hematological complications after neoadjuvant therapy was similar in both groups (Table 2).

**Table 2:** Clinical, radiological response, and adverse effects of neoadjuvant therapy ^#^Modified Takita Grading for Dysphagia; ^*^CTCAE grading version 5; ^a^ Mann-Whitney U test; ^b^ Chi-Square test NACT: neoadjuvant chemotherapy; NACRT: neoadjuvant chemoradiotherapy; IQR: interquartile range; CTCAE: common terminology criteria for adverse events

Parameters	Sub-Category	NACT (n=30)	NACRT (n=33)	p-value
Change in grade of dysphagia^#^, median (IQR)	-	2 (1-3)	2 (1-2)	0.537^a^
Radiological response at primary site, n (%)	Complete Response	2 (6.7)	11 (33.3)	0.013^b^
	Partial Response	18 (60.0)	18 (54.5)	
	Stable Disease	10 (33.3)	4 (12.1)	
	Progressive Disease	0 (0)	0 (0)	
Radiological change in cT, n (%)	0	1 (3.3)	1 (3)	0.104^b^
	1	0 (0)	7 (21.2)	
	2	16 (53.3)	16 (48.5)	
	3	12 (40.0)	8 (24.3)	
	4a	1 (3.3)	1 (3)	
Radiological response at nodal site, n (%)	Complete Response	2 (6.7)	9 (27.3)	0.087^b^
	Partial Response	18 (60.0)	17 (51.5)	
	Stable Disease	10 (33.3)	7 (21.2)	
	Progressive Disease	0 (0)	0 (0)	
Radiological change in cN, n (%)	0	7 (23.3)	14 (42.4)	0.108^b^
	1	23 (76.7)	19 (57.6)	
Anemia^*^, n (%)	Grade 1	26 (86.7)	27 (81.8)	0.598^b^
	Grade 2 or more	4 (13.3)	6 (18.2)	
Neutropenia^*^, n (%)	Grade 1	28 (93.3)	32 (97.0)	0.498^b^
	Grade 2 or more	2 (6.7)	1 (3.0)	
Thrombocytopenia^*^, n (%)	Grade 1	23 (76.7)	28 (84.8)	0.409^b^
	Grade 2	7 (23.3)	5 (15.2)	
Hypoalbuminemia^*^, n (%)	Grade 1	24 (80.0)	26 (78.8)	0.362^b^
	Grade 2 or more	6 (20.0)	7 (21.2)	

Surgical details

All patients underwent MITE with total thoracic lymphadenectomy and laparotomy for gastric conduit formation with abdominal lymphadenectomy. There was a non-significant trend toward longer operative duration in the NACRT group. Intraoperative blood loss, postoperative morbidity, major morbidity (Clavien-Dindo grade IIIA or higher), anastomotic leakage rates, and hospital stay were similar between the two groups (Table 3).

**Table 3:** Perioperative outcomes of minimally invasive esophagectomy ^a ^Mann Whitney U test; ^b^ Chi-Square test; ^*^Clavien-Dindo Grade IIIa or more is considered a major complication NACT: neoadjuvant chemotherapy; NACRT: neoadjuvant chemoradiotherapy; IQR: interquartile range

Parameters	NACT (n=30)	NACRT (n=33)	p-value
Surgical approach, Thoracoscopic / Robotic, n (%)	22 (73.3) / 8 (26.7)	18 (54.5) / 15 (45.5)	0.121^b^
Operative duration (minutes), median (IQR)	420 (360-533)	480 (390-540)	0.282^a^
Blood loss (ml), median (IQR)	200 (150-300)	200 (100-300)	0.683^b^
Anastomosis, handsewn/stapled, n (%)	8 (26.7)/22 (73.3)	9 (27.3)/24 (72.7)	0.957^b^
Postoperative hospital stay (days), median (IQR)	7 (6-8)	6 (6-8)	0.514^a^
Clavien-Dindo grade, < IIIa/IIIa or more*, n (%)	24 (80.0)/6 (20.0)	27 (87.9) / 4 (12.1)	0.454^b^
Chyle leak, n (%)	2 (6.7)	2 (6.1)	0.921^b^
Pulmonary, n (%)	6 (20.0)	2 (6.1)	0.096^b^
Anastomotic leak, n (%)	4 (13.3)	2 (6.1)	0.461^b^
Recurrent laryngeal nerve palsy, n (%)	2 (6.7)	0 (0)	-
90-day mortality, n (%)	4 (13.3)	2 (6.1)	0.326^b^

Histopathological outcomes

The median number of lymph nodes retrieved (21 vs 19) and the R0 resection rate were similar between the NACT and NACRT groups (100% vs 94%). Lymphovascular invasion was greater in the NACT group (36.7% vs 12.1%, p-value - 0.022), whereas perineural invasion was similar (36.7% vs 24.2%, p-value: 0.283). The complete pathological response, tumor regression (grade 0), and downstaging of pT and pN were better in the NACRT group (Table 4).

**Table 4:** Comparison of histopathological and oncological outcomes ^a^ Mann Whitney U test; ^b^ Chi-Square test NACT: neoadjuvant chemotherapy; NACRT: neoadjuvant chemoradiotherapy; IQR: interquartile range; lymph node ratio: number of positive lymph nodes/number of total lymph nodes retrieved

Parameters	Sub-Category	NACT (n=30)	NACRT (n=33)	p-value
Pathological response, n (%)	Complete response	3 (10)	12 (33.3)	0.032^b^
	Near complete response	4 (16.7)	8 (24.2)	
	Partial Response	16 (73.3)	7 (42.4)	
	No Response	7 (23.3)	6 (18.2)	
Tumor regression grade, n (%)	0	1 (3.3)	12 (36.4)	0.004^b^
	1	3 (10.0)	6 (18.2)	
	2	10 (33.3)	6 (18.2)	
	3	16 (53.3)	9 (27.3)	
Pathological (p) T, n (%)	0	3 (10)	14 (42.4)	0.032^b^
	1	5 (16.7)	5 (15.1)	
	2	11 (36.7)	7 (21.2)	
	3	11 (36.7)	7 (21.2)	
Pathological (p) N, n (%)	0	14 (46.7)	26 (78.8)	0.026^b^
	1	7 (23.3)	4 (12.1)	
	2 or more	9 (30)	3 (9.1)	
Lymph node grade, n (%)	No residual tumor	14 (46.7)	24 (72.7)	0.104^b^
	Partial regression	8 (26.7)	4 (12.1)	
	No regression	8 (26.7)	5 (15.2)	
Overall stage, n (%)	0	3 (10.0)	11 (33.3)	-
	I	10 (33.3)	11 (33.3)	
	II	2 (6.7)	6 (18.2)	
	III	13 (43.3)	5 (15.1)	
	IVa	2 (6.7)	0 (0)	
Total lymph nodes retrieved, median (IQR)	-	21 (16-30)	19 (8-26)	0.188^a^
Positive lymph nodes, median (IQR)	-	1 (0-3)	0 (0)	-
Lymph node ratio, median (IQR)	-	0.03 (0-0.14)	0 (0)	-
Resection margin, R0/R1, n (%)	-	30 (100)/0 (0)	31 (93.9)/2 (6.1)	-
Lymphovascular Invasion, n (%)	-	11 (36.7)	4 (12.1)	0.022^b^
Perineural Invasion, n (%)	-	11 (36.7)	8 (24.2)	0.283^b^
Recurrence, n (%)	-	10 (33.3)	10 (30.3)	0.796^b^
Deaths, n (%)	-	12 (40.0)	12 (36.4)	0.766^b^
Recurrence-free survival (months), median (IQR)	-	57	36	0.831^a^

**Figure 2:**
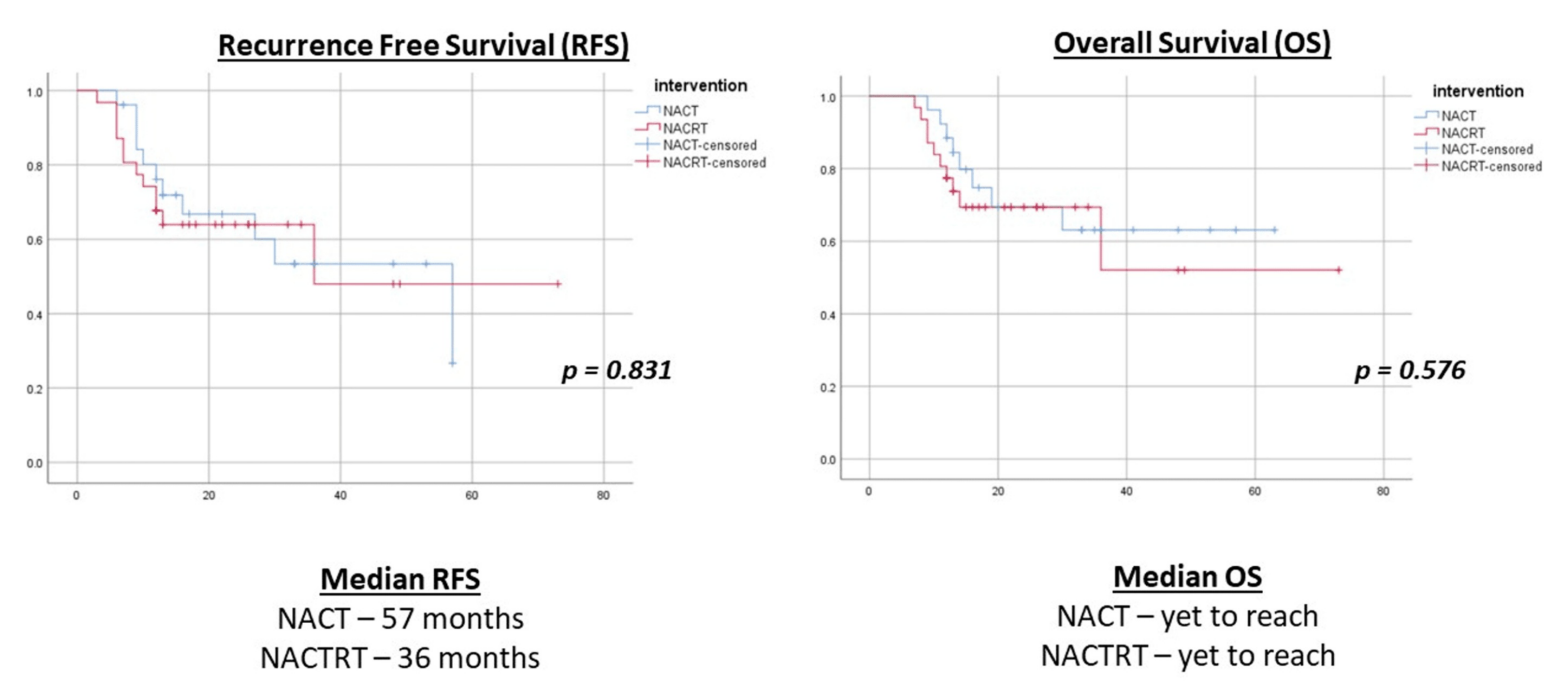
Survival analysis with Kaplan‒Meier survival analysis with the log-rank test between NACT and NACRT NACT: neoadjuvant chemotherapy; NACRT: neoadjuvant chemoradiation

Systemic chemotherapy was given to 7/30 (23.3%) patients in the NACT group and 3/33 (9.1%) patients in the NACRT group after surgery. At a median follow-up of 32.5 (20.75-48) months, 20 patients overall had developed recurrence, and 24 had died. Both the NACT and NACRT groups had similar RFS rates (median 57 vs 36 months) and OS rates (median yet to reach) (Figure 2).

## Discussion

Multimodal therapy has become the optimal treatment strategy in the management of resectable esophageal cancers. The current guidelines entail the administration of some form of neoadjuvant treatment, either chemoradiation or chemotherapy alone, prior to surgical resection [14]. The recent Neo-AEGIS (Neoadjuvant Trial in Adenocarcinoma of the Esophagus and Esophago-Gastric junction International Study) suggested that both chemotherapy and chemoradiotherapy have similar benefits in patients with adenocarcinoma of the esophagus [15]. However, data comparing preoperative chemotherapy with chemoradiotherapy in patients with ESCC are limited. The present study compared the outcomes of NACT and NACRT administration followed by surgery in patients with resectable ESCC.

Numerous NACT regimens have been analyzed to manage ESCC and compare them with radiotherapy; however, the combination of PC, which was part of the CROSS trial, has not been studied in isolation [1,10,16,17]. In the present study, we used the PC regimen and reported that it was well tolerated, similar to the results of the study by Tang et al. [18]. The advantage of this regimen is that we can finish neoadjuvant treatment in three to four weeks and perform surgery once the hematological and nutritional parameters recover, unlike the NACRT regimen, where neoadjuvant treatment is completed in five weeks, and the response takes another five to six weeks.

Neoadjuvant therapy toxicity is always a matter of concern. In the CROSS trial, NACRT was associated with grade 3 or higher side effects in 7% of the patients [1]. Furthermore, the NeoRes (Neoadjuvant Chemotherapy Versus Radiochemotherapy for Cancer of the Esophagus or Cardia) trial revealed greater Clavien-Dindo grade IIIA complications or more complications with NACRT than with NACT [10]. The toxicity of the NACT and NACRT regimens was comparable in the current study, and nearly 90% of patients could complete the neoadjuvant treatment. Furthermore, NACRT has been reported to be associated with a greater incidence of odynophagia, impaired cardiac function after surgery, and chronic cough, which affects the quality of life [2,19].

In discussions of better neoadjuvant strategies for ESCC, an important factor that is often ignored is the quality of surgery. The rate of R0 resection and extent of lymphadenectomy add to the overall patient outcome of both approaches. In our study, the R0 resection rates were 93.9% and 100%, and the median lymph node yields were 19 and 21.5 in the NACT and NACRT groups, respectively. Furthermore, one advantage of our study was that all the surgical procedures were either thoracoscopic or robot-assisted esophagectomy with two-field lymphadenectomy. The magnified view of the thoracoscope improves the dissection of both primary and lymph nodes and helps perform radical resection with decreased postoperative morbidity. However, the surgical technique was heterogeneous in the CROSS and NeoRes trials, where some patients underwent transhiatal esophagectomy [1,10].

The rationale behind the consideration of NACT over NACRT is to avoid overtreatment of local disease with radiation as well as surgery. In our study, the systemic treatment received in both arms was the same, but the NACRT group received two local treatments (surgery and radiation). Hence, if good-quality surgery with adequate lymphadenectomy is feasible, then one might consider avoiding radiation among these patients. Furthermore, a high pCR rate, a finding in this study, has often been quoted as an argument supporting NACRT over NACT. However, pCR means good local control, but it might not mean a complete cure or improved cancer survival, as distant metastases can still occur. This finding is observed in multiple studies, including this study, which compared NACT and NACRT, where a higher pCR failed to lead to higher RFS or OS [10,15,17,18].

The operative duration of the thoracic phase was slightly greater in the NACRT group than in the non-NACRT group, indicating some surgical difficulty due to RT-associated inflammation and fibrosis. Although we cannot prospectively monitor the degree of surgical difficulty, it needs to be analyzed in future studies. However, the NACRT arm was not associated with increased postoperative complications or mortality, similar to the conclusions of recent randomized controlled trials (RCTs) and a meta-analysis [10,17,20].

Postoperative complications and hospital stays were comparable between the two groups. This might be because the MITE was performed by the same experienced surgical team with standardized steps and a perioperative enhanced recovery after the surgery protocol [21,22]. Furthermore, the NeoRes trial revealed that the incidence of surgical or nonsurgical complications and 90-day mortality rates were not significantly different between the preoperative chemotherapy and chemoradiation groups (p-values = 0.69 and 0.13, respectively) [10,19].

In the present study, the radiological and pathological complete response (pCR) rates were significantly higher in the NACRT group than in the NACT group. However, after a median follow-up of 32.5 months, the recurrence rate was similar between the NACT and NACRT groups (33.3% vs 30.3%). Furthermore, there was a non-significant trend toward better RFS in the NACT group (median RFS 57 months vs 36 months, p = 0.831), with no significant difference in OS (p = 0.576). Similarly, Miller et al. reported a higher median OS (40.8 vs 36 months) and a similar five-year OS (43% vs. 39%) with NACT vs. NACRT for ESCC [23]. The JCOG1109 (NExT) study for cStage II or III ESCC compared two NACT regimens with NACRT. They reported higher OS with triplet NACT and no superiority of NACRT over doublet NACT [17]. Finally, an RCT by Tang et al. reported that NACRT followed by MITE for locally advanced ESCC had a comparable three-year survival rate (64.1% for NACRT vs 54.9% for NACT, p = 0.28) and RFS (49.2 vs 50.2 months, p = 0.75) with NACT, despite having a significantly higher pCR (27.7% vs 2.9%, p <0.001) and primary tumor regression grade (p <0.001) in the NACRT group [18]. Hence, the outcomes observed in the present study and the studies mentioned above imply that despite a greater tumor response in the NACRT group, this response did not translate into increased OS and RFS.

The treatment of ESCC further evolved after the introduction of immunotherapy agents. The CheckMate 577 trial established the role of adjuvant nivolumab in esophageal cancer patients who underwent surgery after NACRT [24]. Encouraged by these results, the FRONTiER study aimed to evaluate the role of nivolumab combined with chemotherapy in the neoadjuvant setting [25]. The appropriate approach for treating locally advanced ESCC will evolve with neoadjuvant and adjuvant immunotherapy combined with chemotherapy.

Our study has a few limitations. First, we could not randomize the allotment to the NACT and NACRT groups because of logistic problems arising from the COVID-19 pandemic and the availability of radiotherapy slots. Nevertheless, we could perform a quasi-experimental study with allocation based on patient choice. Second, our sample size was small and may be underpowered to interpret differences in many variables. However, this study is the first prospective report comparing NACT and NACRT in the MITE era in Indian patients. Furthermore, the findings of this study should pave the way for future large RCTs using PC-based NACT regimens.

## Conclusions

NACT using the PC regimen is well tolerated in Indian patients and has non-inferior oncological outcomes compared with the CROSS NACRT regimen despite inferior pathological responses. The additive role of radiation appears minimal when good local control can be achieved with high-quality surgery and radical two-field lymphadenectomy. NACT can be considered a feasible strategy compared with NACRT if radiotherapy is contraindicated or unavailable in certain situations.
